# The anatomy of a nucleus: As revealed by chromosome painting

**DOI:** 10.1371/journal.pgen.1007445

**Published:** 2018-07-12

**Authors:** Brian D. Slaughter, R. Scott Hawley

**Affiliations:** 1 Stowers Institute for Medical Research, Kansas City, Missouri, United States of America; 2 Department of Molecular and Integrative Physiology, University of Kansas Medical Center, Kansas City, Kansas, United States of America; Geisel School of Medicine at Dartmouth, UNITED STATES

Many factors play a role in determining which genes are active or inactive in cells at various stages of the cell cycle. The spatial organization of the genome is one such factor, encompassing things such as topologically associated domains (TADs), looping of enhancers toward or away from targets, and the interaction of loci with the nuclear envelope or nucleolus [1]. Identifying and understanding the multiplicity of interactions along and between the chromosomes will allow us to answer many challenging questions in biology, such as how structural variation in the genome affects transcription. In this issue of *PLOS Genetics*, Joyce and colleagues use Oligopaint to mark and examine the topology of *Drosophila* chromosomes and uncover an interesting role for condensin II in organizing the genome [2].

Our prior understanding of inter- and intrachromosomal interactions was uncovered using several different technologies. One such technology is chromosome conformation capture (3C), which links nearby genomic regions that are then identified using genome sequencing. This method is ideal for identifying short-range looping, chromosome territories, and TADs [3]. However, with a few single-cell exceptions [4], most 3C data include a variety of different cell types at potentially various stages of the cell cycle and thus fail to reveal cell-to-cell heterogeneity. A complementary technique for examining genome organization is DNA fluorescence in situ hybridization (FISH). DNA FISH of 3 to 5 loci can provide information about the distribution and distance between selected loci within the nucleus with exquisite detail; however, it does not provide enough information to uncover genome-wide organization. Although these 2 techniques provide related information, one must use caution when directly comparing them [5,6]. The missing link between these 2 methods involves imaging all chromosomes at single-cell resolution to gain information about overall organization, including which chromosomes are adjacent to or secluded from one another. This technique has been accomplished using DNA-painting probes, such as SKY paint [7,8].

In search of more freedom in probe design, including more control over probe coverage level and probe length, biologists have designed methods to use high-number custom oligo sequences (with unique barcodes on groups of oligos) followed by amplification methods to generate flexible DNA FISH probe libraries [9]. These libraries can be customized to paint whole chromosomes or to mark specific chromosomal identities, such as transcriptionally active, inactive, or Polycomb-repressed regions, and can be imaged with super-resolution microscopy to find the precise location of these areas within the nucleus [10,11]. Protocols and tools are available for large-scale probe design and use [12,13,14], putting the power of this technique into the hands of a much larger number of biological researchers.

**Fig 1:**
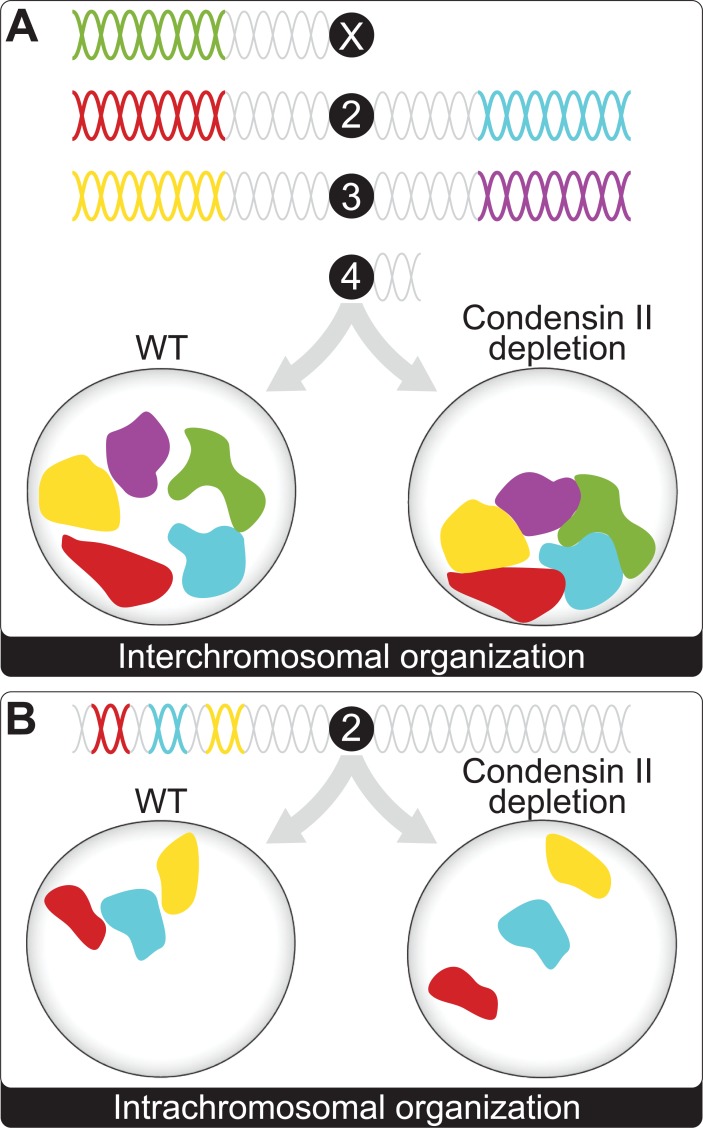
Oligopainting of the *Drosophila* genome and the effect of condensin II depletion. (A) Custom Oligopaint probe libraries were designed to mark the nonrepetitive regions of the *Drosophila* genome. The extent of chromosome intermixing was calculated and found to increase upon condensin II knockdown. (B) The Oligopaint library was modified to mark 3 regions of 1 arm of chromosome 2. Condensin II knockdown led to an increase in an “open” configuration of the arm.

By using these custom oligo probe groups in combination with automated computational methods, one can search both for features of large-scale chromosome organization and for unique features of single cells that may be correlated back to gene expression or aberrant cellular function. The paper by Joyce and colleagues makes 3 key contributions to our understanding of genome organization in *Drosophila*. First, they designed and validated an Oligopaint library that marks the entire nonrepetitive portion of the *Drosophila* genome, with the X, 2nd, and 3rd chromosomes marked with unique colors (Fig 1). Second, they used this Oligopaint library to provide exquisite detail on how chromosome territories are conserved in different *Drosophila* cell types.

Third, the authors focused on the role of condensin and cohesin in the formation of chromosome territories. The structural maintenance of chromosome (SMC) complexes are known to play a role in the spatial organization of the genome and correspondingly affect gene expression [15,16,17,18,19,20]. Interestingly, while they found little change in large-scale genome organization or chromosome intermixing upon cohesin or condensin I depletion, the authors found that condensin II (Cap-H2) depletion leads to increased intermixing of chromosomes, effectively “mixing” the territories (Fig 1A). Overexpression of condensin II decreased this overlap between chromosomes. Taking advantage of the flexibility in labeling specific oligo probe groups, the authors also used a 3-color approach across a single chromosome, 2L, to specifically look at intrachromosomal packing and territory arrangement. In their analysis, they found that condensin II depletion led to a more open configuration, in effect reducing interactions between different domains of the same chromosome (Fig 1B). Conversely, condensin II overexpression increased the frequency of closed configurations. The observations presented by Joyce and colleagues allow a much deeper understanding of the role that condensin II plays in both interchromosomal and intrachromosomal organization and in mediating the spatial overlap of chromosome domains.

There are several additional observations made by Joyce and colleagues that will undoubtedly guide future research. While conserved features of genome organization and chromosome territories were observed, a small percentage of cells displayed pairwise overlap far from the mean, with large disrupted territories or a lack of discrete territories. This subgroup of cells wasn’t the focus of this study, but these outliers could lead to a new understanding of the importance of territories or the factors that control them. Do these cells also display their own conserved features of genome organization? Do they display aberrant gene expression?

Finally, the authors demonstrate that the general layout of chromosome territories appears to be conserved in different *Drosophila* cell types. Perhaps there are general principles that have not previously been appreciated, and thus as Oligopainting becomes a more generally used technique, the field may build up “consensus” territories between cells from different organisms. If vast differences are observed between organisms, why? And are these potential differences related to differences in gene expression, leading to differences in cell structure, function, or fate?

It will be crucial for future studies to combine methods of verification of chromosome territories, chromosome looping, etc., with the expression of given genes. Indeed, work has already begun with the use of Oligopaint libraries designed to mark regions that are transcriptionally active, inactive, and Polycomb repressed [11]. Such libraries could be combined with Oligopaint probes to certain areas of the genome. Complementary methods also include the combination of live-cell dCas9 to target genomic loci, followed by cell fixation and RNA FISH to allow for direct visual interrogation of the relationship of changes in small-range interactions between chromosomes and gene bursting [21].

Multiple lines of evidence point toward complex and multiplexed mechanisms of control of short-range and long-range interactions of chromosome territories, in addition to mechanisms that control localization within the nucleus. While short-term interactions are being diagnosed with 3C methods, organizational principles that either span larger distances or are characterized by exclusion (rather than inclusion) of a pair of areas may only be diagnosed with methods such as Oligopainting. Merging all of this data toward an integrative model that explains differences in different systems will not be easy and will require a great amount of data. Continued collaboration among genomicists, geneticists, computer scientists, and biophysicists will be essential [1]. Consider also the ongoing search for rare long-range interactions in those cells that do not fit the average, and it is easy to see how Oligopaint approaches will be a necessary part of our biological toolkit.
